# Phage-mediated resolution of genetic conflict alters the evolutionary trajectory of *Pseudomonas aeruginosa* lysogens

**DOI:** 10.1128/msystems.00801-24

**Published:** 2024-08-21

**Authors:** Laura C. Suttenfield, Zoi Rapti, Jayadevi H. Chandrashekhar, Amelia C. Steinlein, Juan Cristobal Vera, Ted Kim, Rachel J. Whitaker

**Affiliations:** 1 Department of Microbiology, School of Molecular and Cellular Biology, University of Illinois at Urbana-Champaign, Urbana, Illinois, USA; 2 Carl R. Woese Institute for Genomic Biology, University of Illinois at Urbana-Champaign, Urbana, Illinois, USA; 3 Department of Mathematics, University of Illinois at Urbana-Champaign, Urbana, Illinois, USA; University of California Irvine, Irvine, California, USA

**Keywords:** lysogen, spontaneous induction, evolution, CRISPR, transposable phages

## Abstract

The opportunistic human pathogen *Pseudomonas aeruginosa* is naturally infected by a large class of temperate, transposable, Mu-like phages. We examined the genotypic and phenotypic diversity of *P. aeruginosa* PA14 lysogen populations as they resolve clustered regularly interspaced short palindromic repeat (CRISPR) autoimmunity, mediated by an imperfect CRISPR match to the Mu-like DMS3 prophage. After 12 days of evolution, we measured a decrease in spontaneous induction in both exponential and stationary phase growth. Co-existing variation in spontaneous induction rates in the exponential phase depended on the way the coexisting strains resolved genetic conflict. Multiple mutational modes to resolve genetic conflict between host and phage resulted in coexistence in evolved populations of single lysogens that maintained CRISPR immunity to other phages and polylysogens that lost immunity completely. This work highlights a new dimension of the role of lysogenic phages in the evolution of their hosts.

**IMPORTANCE:**

The chronic opportunistic multi-drug-resistant pathogen *Pseudomonas aeruginosa* is persistently infected by temperate phages. We assess the contribution of temperate phage infection to the evolution of the clinically relevant strain UCBPP-PA14. We found that a low level of clustered regularly interspaced short palindromic repeat (CRISPR)-mediated self-targeting resulted in polylysogeny evolution and large genome rearrangements in lysogens; we also found extensive diversification in CRISPR spacers and *cas* genes. These genomic modifications resulted in decreased spontaneous induction in both exponential and stationary phase growth, increasing lysogen fitness. This work shows the importance of considering latent phage infection in characterizing the evolution of bacterial populations.

## INTRODUCTION

Cystic fibrosis (CF) is a genetic disorder that makes patients vulnerable to respiratory infections by environmental opportunistic pathogens like *Pseudomonas aeruginosa. Pseudomonas aeruginosa* is a common human pathogen whose increase in multi-drug antibiotic resistance has made it the focus of targeted phage therapy (1). In chronic *Pseudomonas* infections of CF patients, it is common to find that all isolates track their origin to a single ancestral genotype (2–8). Diversity of *P. aeruginosa* strains emerges from this ancestor through *de novo* mutations generated by mutation and hypermutation genotypes (9), recombination (9, 10), and large deletions (11–13).

*Pseudomonas aeruginosa* is commonly infected by latent bacterial viruses (phages) when colonizing CF patients (3, 9, 14–19). Canonically, temperate and chronic phages can act as both sources of genetic novelty (20) and as potential assassins that can be induced to kill their hosts (21, 22). In the lung environment, the presence of antibiotics and reactive oxygen species (23–25) may act as inducing agents for phage (21, 26–29). Bacterial lysis through phage induction is hypothesized to help control bacterial growth in the lung (21) and may potentially be used in synergy with antibiotics (30, 31). Additionally, lysogeny has been shown to co-occur with host genome rearrangements in chronically infecting *Staphylococcus aureus* (32–35) and *Streptococcus pyogenes* (36). However, if and how lysogeny alters the evolution of the host genome remains unclear.

Mu-like transposable phages are a diverse family of phages that infect an equally diverse range of bacteria (37). Upon infection of the host, Mu-like phages integrate into the host chromosome through a conservative (cut-and-paste) transposition step that occurs with low sequence preference (38–40). Lytic replication occurs via replicative (copy-and-paste) transposition around the genome (41), which occurs 50–100 times and terminates in headful packaging into the virion. In lysogeny, which is established in approximately 10% of infections, a low-specificity insertion can increase the variation available to natural selection in *P. aeruginosa* populations through loss-of-function mutations (42–44). *P. aeruginosa* lysogens have previously been found to have high viral titers in culture (26, 45). Due to the nature of the chemistry that governs the transposition reaction, these insertions may also cause structural rearrangements such as deletions (46).

CRISPR-Cas (clustered regularly interspaced short palindromic repeats and CRISPR-associated genes) is a bacterial and archaeal adaptive immune system that incorporates foreign DNA fragments into an array as a spacer and subsequently targets any piece of invading DNA that is complementary to the spacer (the protospacer) (47, 48). Mu-like temperate phages are the most commonly targeted phages by CRISPR-Cas in *Pseudomonas aeruginosa* (49). Phage DMS3, a member of this group, was recovered from a *P. aeruginosa* CF isolate and infects the type strain UCBPP-PA14 (14). DMS3 lysogens inhibit quorum sensing and pilus formation (50). PA14 contains a Type 1-F CRISPR system with a partial spacer match to DMS3 (51). This spacer has five mismatches to the phage protospacer, which is not sufficient to mediate immunity to the phage but leads to genetic conflict in DMS3 lysogens. This degenerate protospacer-spacer mismatch between DMS3 and PA14 targets the PA14 chromosome, causing enough DNA damage to stimulate the SOS response, which leads to the expression of pyocin genes, cell death, and limitation of biofilm formation (52). Lysogens arising from PA14 cultures infected with free DMS3 virions evolved to have a lower spontaneous induction in the stationary phase and lost their CRISPR systems over a 7-day evolutionary period. This was suggested to resolve genetic conflict caused by CRISPR self-targeting (immunopathology), a phenomenon that is predicted to be common in bacteria with Type 1 CRISPR systems and temperate phages (44).

Here, we assess the contribution of CRISPR-mediated genetic conflict between host and temperate phage to the evolution of *Pseudomonas* by analyzing evolved lysogen and non-lysogen populations. We show that selection to resolve genetic conflict alters the evolutionary landscape of lysogen populations and that spontaneous induction rates correlate with the type of conflict resolution. Experimental work combined with genomic analysis demonstrates that transposable phages are a major source of variation beyond mutation that impacts the evolutionary direction of *P. aeruginosa* lysogens.

## MATERIALS AND METHODS

### Experimental evolution

To establish the contribution of phages to host genome evolution, we evolved the uninfected laboratory strain UCBPP-PA14 (53) and the established lysogen Lys2 (51) for 12 days by serial transfer. Our strains are listed in Table S1. Lys2 is derived from PA14 and contains DMS3, which mediated a ~20 kb host deletion from 806,169 to 826,108 bp on our PA14 reference chromosome, and a single A < G nonsynonymous point mutation at position 4,755,306 in a hypothetical protein (deleted genes are listed in Table S2). For each strain, we randomly selected three colonies and grew up independent overnight cultures in LB (10 g tryptone, 5 g yeast extract, and 5 g sodium chloride per liter of water). We subcultured the cells and normalized them to an OD_600_ of 0.2 in 25 mL microcosms in three parallel 250 mL flasks. We serially passaged triplicate cultures with daily 1:100 transfers for 12 days, with shaking and at 37°C, for approximately 72 bacterial generations. At the end of the experiment, we colony-purified six colonies isolated from each replicate and six colonies from the Lys2 ancestor stock for further analysis. Six isolates from each uninfected population were also sequenced, and the data are presented in Fig. S1.

### One-step growth curves

In order to calculate the burst size of DMS3, we performed one-step growth curves (54). Overnight, stationary phase PA14 cultures were diluted 1:100 and grown in LB until they reached an OD of 0.5. Phage was added to these cultures at a 1:10 volume ratio, for a final MOI of 0.01, mixed well, and incubated at 37°C for 5 minutes. In order to calculate adsorption, a fraction of the sample volume was immediately spun down for 5 minutes at 30,000 rpm, and the supernatant was stored with 10% (vol/vol) chloroform to quantify the remaining free phage. To begin the one-step growth curve, the remainder of the samples were added to a pre-warmed medium at a 1:100 ratio to stop adsorption and grown on a roller drum at 37°C for the next 2.5 hours. Samples were taken every 10 minutes and mixed with 10% (vol/vol) of chloroform for later quantification of PFUs.

We calculated burst size with the following equation: the total phage produced (the difference between the average PFUs before and after the burst) was divided by the number of cells that were infected (estimated by the number of adsorbed phages multiplied by the estimated number of cells that proceeded through lysis). Our measurements of an 8% lysogenization frequency, estimated by spot-on-lawn assay, corresponded to measures in reference (55) (data not shown).


$$\beta =\frac{p_{2}-p_{1}}{c_{i}*\left(1-l\right)}.$$

Here, *β* is burst size; *p*_2_ and *p*_1_ are the second and first plateaus, respectively; $c_{i}$ is the number of infected cells; and l is the lysogenization frequency. The burst size of DMS3 is 41.8 ± 8.4 phages per lysed cell (Fig. S2). All PFUs were enumerated by spotting the phage-containing fraction on 0.5% double agar overlay plates.

### Spontaneous induction measurements

Because multiple inputs could contribute to a higher raw PFU value in the stationary phase and because the stationary phase itself could be an inducing condition for some viruses (including phage Mu) (56), we chose to measure spontaneous induction separately in both exponential and stationary phase, which necessitated the normalization of PFU values with the CFU values. This was also a desirable method for controlling whether increased growth rate was responsible for increased PFU values.

Growth curves were started from overnight cultures of replicate purified isolates. We washed cultures three times by resuspension in fresh LB media, normalized the OD to 0.2, and diluted them 1,000-fold. Time points were taken at 0 and 2–7 hours to capture exponential phase growth and plated for both CFUs and PFUs. Samples were incubated at 37°C on a roller drum. We measured spontaneous induction (q) by taking the difference in the number of viral particles released by cells growing in the exponential phase and normalizing by the estimated burst size, the average growth of the culture, and the total time the culture grew.


$$q=\frac{\Delta V}{\left(\beta \times \Delta t\times C\right)}.$$

Here, q is spontaneous induction and has units of burst cells per time; ∆V is the total increase in virion particles; β is the burst size; ∆t is the change in time; C is the average amount of cells in the culture. To account for exponential growth, all calculations were based on the linear regression of the log_10_-transformed ∆V and C values.

We also calculated spontaneous induction based on calculations in reference (57). This paper approximated spontaneous induction at each time point with this formula: $q=\frac{V}{\beta C}$ ; here, V is the number of virion particles at that time; β is the burst size; and C is the average amount of CFUs at that time. These methods produced qualitatively similar results (Fig. S3).

To measure induction in the stationary phase, we began growth curves in the same way as the exponential phase measurements. Time points were taken at 0, 10, 12, 15, and 18 hours and plated for both CFUs and PFUs. To calculate the rate of spontaneous induction, we used the same formula as above, except with un-regressed logged values.

### Genome sequencing

In order to create a viral reference, DMS3 DNA was extracted from filtered Lys2 overnight supernatant using the Phage DNA Isolation Kit (Norgen Biotek Corp, Cat #: 46800) following the manufacturer’s instructions. Libraries were prepared with a Biomek 4000 liquid handler (Beckman-Coulter). We quantitated libraries with a Qubit fluorometer (Life Technologies Corporation, REF #: Q32866). Libraries were pooled and submitted for 2 × 250 paired-end sequencing by the Roy J. Carver Biotechnology Center at the University of Illinois Urbana-Champaign with an Illumina NovaSeq 6000. We received ~3.8 million reads with about 100× coverage.

We inoculated evolved isolates and ancestral controls in 2 mL deep well plates in LB and grew them overnight. We extracted gDNA with the Beckman-Coulter gDNA extraction kit as above using the Nextera Flex Library Preparation Kit (Illumina). We quantitated libraries with a Qubit fluorometer (Life Technologies Corporation, REF #: Q32866). Libraries were pooled and submitted for 2 × 250 paired-end sequencing by the Roy J. Carver Biotechnology Center at the University of Illinois Urbana-Champaign with an Illumina NovaSeq 6000. We received an average of about 5 million reads per genome. All raw reads are available on the NCBI database under BioProject number PRJNA1021667.

### Genome analysis

We ran a custom QC pipeline on our raw FASTQ reads, available on Github (http://www.github.com/igoh-illinois). Briefly, the Illumina adaptor sequences were trimmed using TrimmomaticPE version 0. Read quality was checked with FastQC version 0.11.9 (options: --noextract -k 5 -f fastq). Reads were aligned using BWA-MEM (58) with default options to a two-contig reference genome containing both our reference PA14 sequence and our reference DMS3 sequence. To identify chromosomal mutations, we ran Breseq (59) with default settings on the trimmed and quality-controlled reads. SAM files were checked manually in both IGV version 2.12.3 (60) and Tablet version 1.21.02.08 (61).

To identify insertion sites of transposable phage, we ran a second, custom pipeline, available on Github (http://www.github.com/igoh-illinois). The pipeline identifies insertion sites at nucleotide resolution by identifying reads that map to both the host and viral chromosome (“split” reads). It further identifies insertion sites by finding reads that have been split on either side of a 5-bp window, creating a small overlapping region when mapped back to the host genome. This is the result of a 5-bp duplication, which is characteristic of Mu and Mu-like phage transposition (62, 63).

While manually verifying the phage insertion sites in IGV, we found putative large duplicated regions of the host chromosome. To verify these duplicated regions, we used the depth command in Samtools (64) to find the number of reads that covered each position in the genome and graphed this using RStudio (R version 4.3.1) (65). In order to assess the protein content of deleted and duplicated regions, FASTA files of sequence of the reference PA14 genome were given to the eggNOG-mapper-v2 pipeline (settings: genomic data; default options) (66).

We used the CRISPR Comparison Tool Kit (CCTK version 1.0.0) to identify and compare CRISPR arrays of the lysogens (settings: crisprdiff; default options) (67).

### Mitomycin C induction experiments

Single colonies of the strains of interest were inoculated in LB, grown overnight at 37°C on a roller drum, and subcultured until they reached an OD of 0.5. Cultures were normalized, split, and incubated with or without 0.5 µg/µL mitomycin C for 3.5 hours, after which CFUs and PFUs were enumerated.

### Model information

We use a compartmental model based on a system of ordinary differential equations. There are six lysogen compartments, each representing a lysogen characterized by a distinct rate of spontaneous induction, but otherwise being identical. Lysogens are induced at their associated rates and transition into the lytic state, which is followed by phage production and bursting. The model is along the lines of the ones presented and analyzed in our previous works (31, 68, 69). All lysogens are assumed to grow at the same rate, and the total bacterial population grows logistically. The model equations read:


$$\frac{dL_{i}}{dt}=rL_{i}\left(1-\sum_{j=1}^{6}\left(L_{j}+I_{j}\right)\right)-q_{i}L_{i},$$

$$\frac{dI_{i}}{dt}=-\delta I_{i}+q_{i}L_{i},$$

$$\frac{dV_{i}}{dt}=\beta \delta I_{i}-\mu V_{i}.$$

We have partially non-dimensionalized the model so that 1 time unit in the simulations corresponds to about 50 minutes. The lysogeny growth rate is denoted by r, the spontaneous induction rates by $q_{i},i=1,\ldots ,6$, the rate of phage production by δ, the burst size is by β, and the rate of viral degradation by μ. The first two equations are decoupled from the last one describing the phage (since we do not consider superinfections in this model). Therefore, the dynamics of the bacterial compartments resemble those of generalized Lotka-Volterra competition.

### Statistical measures

Data visualization and statistical analyses were performed in R version 4.3.1 (65) using the packages tidyverse version 2.0.0 (70), car version 3.1-2 (71), rstatix version 0.7.2, and emmeans version 1.8.6.

## RESULTS

### Evolution of a self-targeting lysogen results in decreased spontaneous induction in exponential and stationary phases

**Fig 1:**
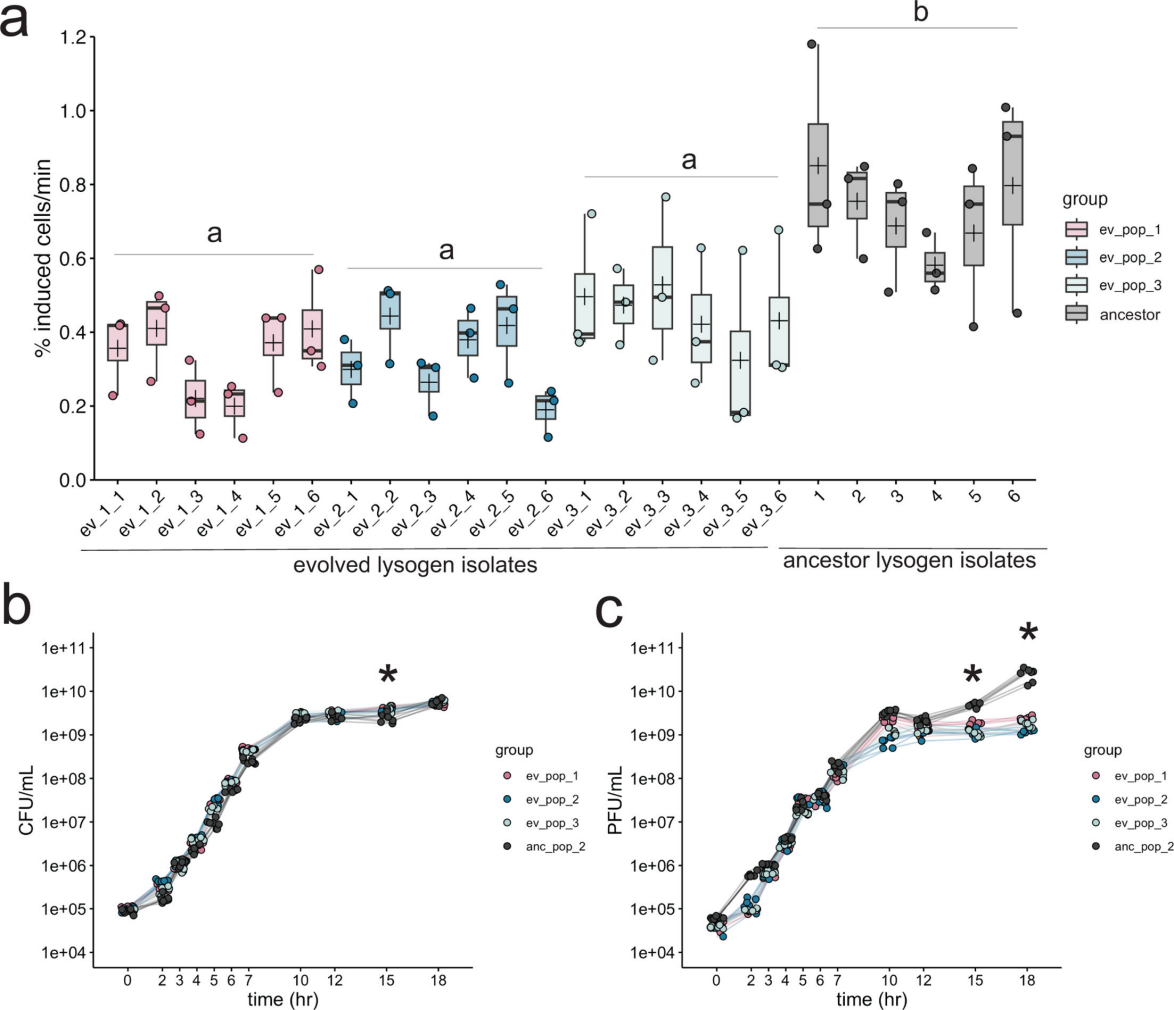
Experimental evolution results in lowered lysogen spontaneous induction. (a) Spontaneous induction was measured in the exponential phase in six individual isolates from each of three evolved lysogen replicates (ev_pop_1, pink, ev_pop_2, blue, and ev_pop_3, light blue) and the ancestral strain Lys2 (anc_pop_2, gray). Points are the means of three technical replicates. Bars in the boxplots represent the median; crosses represent the means. Upper and lower bounds of the box are the upper and lower interquartile ranges. Significance was tested with an ANOVA (*F*_3,68_ = 23.27, *P*-value = 1.78e-10). Letters indicate significance; groups with different letters have a *P*-value < 0.05; groups with the same or overlapping letters have a *P*-value of >0.05. (b) Bacterial growth curve of all isolates through 18 hours, measured by CFU. (c) PFUs sampled through growth curve of all isolates (except the non-lysogenic WT PA14). In panels b and c, stationary phase was measured in separate experiments, as indicated by the line breaks between 7 and 10 hours. Points represent the mean of three biological replicates. Asterisks represent a significant difference between the ancestral and evolved populations. Significance was calculated with a Mann-Whitney *U* test per time point.

After 12 days and ~72 generations of exponential growth in rich media, we found that the spontaneous induction of DMS3 lysogens was significantly reduced compared to ancestral isolates in the exponential phase (Fig. 1; ANOVA, *F*_3,68_ = 16.7, *P* < 1e-8). Isolate induction rates within and between experimental replicates ranged from 0.1% to 0.71% of the culture, while the induction rates of ancestral PA14 lysogen (Lys2) isolates ranged from 0.38% to 1.1% of the culture (Fig. 1a). Significant differences were robust to the use of other metrics to estimate spontaneous induction (57) (see Fig. S3). Spontaneous induction was also significantly decreased in the stationary phase for all evolved lysogens (Fig. S4; ANOVA, *F*_3,109_ = 86.98, *P* < 2.2e-16), although compared to the exponential phase, induction was very reduced in stationary phase overall, indicating that the majority of spontaneous induction takes place in the exponential phase in DMS3 lysogens.

### Lysogen populations maintain diversity in CRISPR presence and function

**Fig 2:**
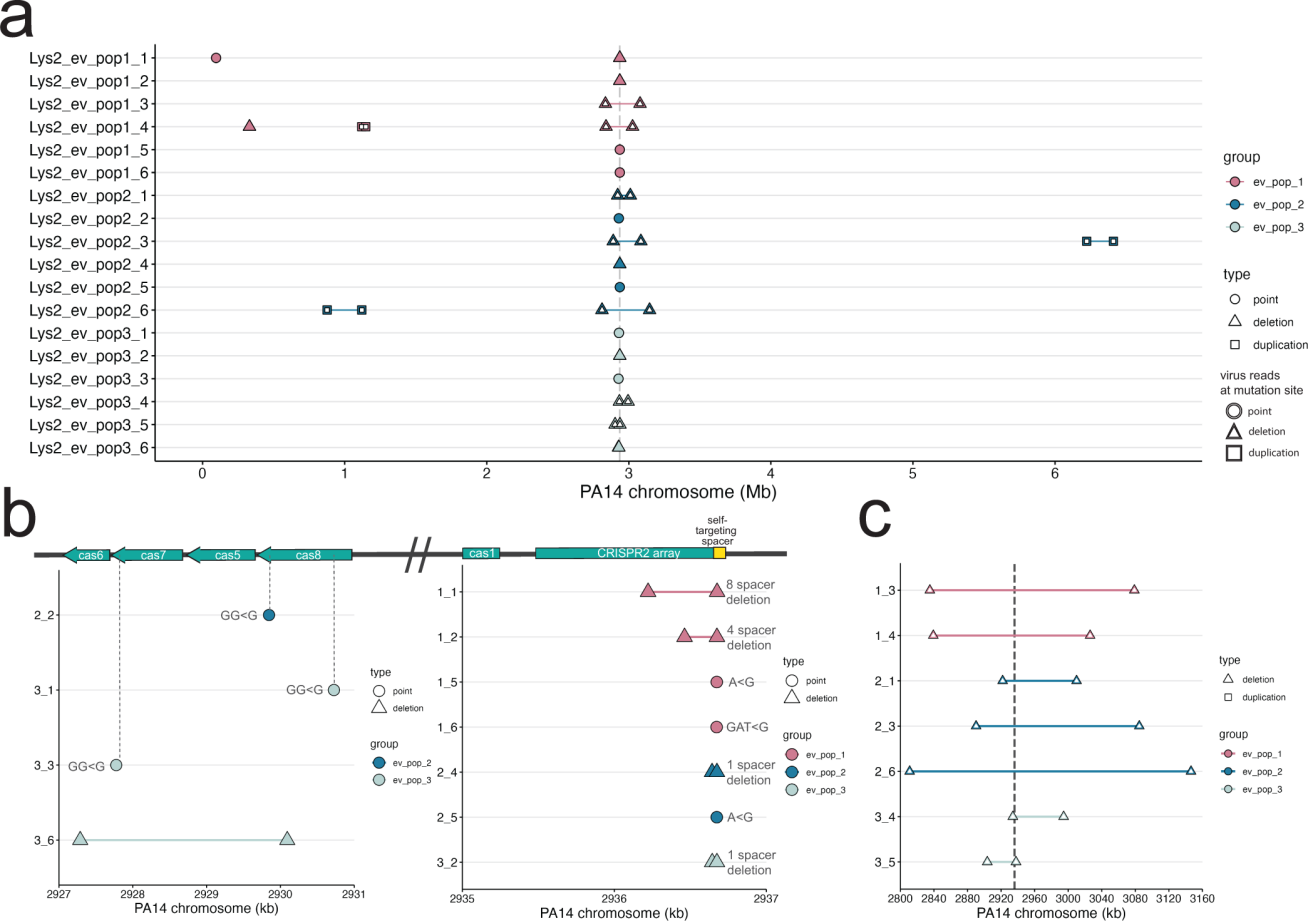
Mutations in infected evolved strains are distinct from uninfected evolved strains. (a) Graph of mutations found in all infected evolved samples. (b) Close-up of point mutations and small deletions in infected evolved strains. The majority are found in the self-targeting spacer 1 in the second CRISPR array. Gray dashed lines indicate the location in the genes. (c) Close-up of large deletions in infected evolved strains. Gray dashed double lines indicate the boundaries of the CRISPR-Cas region. In panels a–c, the *y*-axis indicates sample ID; *x*-axis indicates the position on the PA14 reference chromosome. Points represent point mutations; triangles spanned by a segment represent deletions of the spanned region; squares spanned by a segment represent duplications of the spanned region. White fill indicates a mutation that was caused by a virus.

Sequencing of lysogens from each population showed that lysogens evolved the CRISPR locus through a combination of mutation and large deletions and other structural variants (Table 1; Fig. 2). These mutations did not overlap with uninfected evolved populations, which exhibited point mutations in flagellar and quorum sensing loci (Fig. S1; Table S3), typical of other laboratory evolution experiments (72). Thirty-nine percentage (7/18) of isolates mutated or deleted spacer 1 in the CRISPR2 array, which contains the mismatched spacer that targets the integrated DMS3 (Fig. 2b; Fig. S5). Two mutations occurred in parallel between two different replicate populations in the evolved lysogen treatment: an A to G point mutation in the seed region of the self-targeting spacer, and an exact deletion of the self-targeting spacer and its upstream repeat (Fig. 2b; Fig. S6). Twenty-two percentage (4/18) of isolates had disruptions (three independent frameshift mutations and a small deletion) within the *cas* genes *cas7* and *cas8*, which form part of the complex that mediates interference in *P. aeruginosa* (73). Of these three frameshift mutations*,* one led to the predicted loss of the *cas7* RNA-binding domain, and two are predicted to interfere with the cas3 recruitment or interaction domain of cas8 (Fig. 2b; Table 1). One strain was recovered with a 3-kb deletion in the *cas* gene region that spans *cas5* (also part of the interference complex) and *cas7*. While each strain with mutations and indels in the CRISPR array likely no longer targets the DMS3 prophage, they maintain CRISPR function. We confirmed that the evolved lysogens did not acquire new spacers (Fig. S6).

**TABLE 1:** Description of mutations recovered in evolved lysogens

Group	Sample ID	Mutated region	Description	Due to phage	Location on PA14-REF chromosome
ev_pop1	ev_pop1_1	CRISPR2 sp1-8	Deletion	No	2,936,222–2,936,676
ev_pop1	ev_pop1_1	*tssK*	Type 6 secretion system baseplate gene; nonsynonymous	No	94,370
ev_pop1	ev_pop1_2	CRISPR2 sp1-4	Deletion	No	2,936,462–2,936,675
ev_pop1	ev_pop1_3	∆ 243,737 bp	Includes CRISPR region	Yes	2,835,111–3,078,848
ev_pop1	ev_pop1_4	∆ 186,866 bp	Includes CRISPR region	Yes	2,839,227–3,026,093
ev_pop1	ev_pop1_4	27,769 bp	Duplicated region	Yes	1,120,309–1,148,078
ev_pop1	ev_pop1_4	*cysT*	Sulfate transport protein; (CAG)_4→3_	No	329,528
ev_pop1	ev_pop1_5	CRISPR2 sp1	A < G mutation in the second nucleotide	No	2,936,674
ev_pop1	ev_pop1_6	CRISPR2 sp1	GAT < G deletion of first and second nucleotide	No	2,936,673
ev_pop2	ev_pop2_1	∆ 88,123 bp	Includes CRISPR region	Yes	2,921,860
ev_pop2	ev_pop2_2	*cas8*	Frameshift (predicted loss of C-term helical bundle region)	No	2,929,846
ev_pop2	ev_pop2_3	∆ 194,512 bp	Includes CRISPR region	Yes	2,890,191–3,084,703
ev_pop2	ev_pop2_3	187,901 bp	Duplicated region	Yes	6,222,744–6,410,645
ev_pop2	ev_pop2_4	CRISPR2 sp1	Deletion	No	2,936,643–2,936,675
ev_pop2	ev_pop2_5	CRISPR2 sp1	A < G mutation in the second nucleotide	No	2,936,674
ev_pop2	ev_pop2_6	∆ 335,331 bp	Includes CRISPR region	Yes	2,811,042–3,146,373
ev_pop2	ev_pop2_6	244,019 bp	Duplicated region	Yes	876,284–1,120,303
ev_pop2	ev_pop2_6	Intergenic region	(GCCAAC)_11→8_	No	3,792,568
ev_pop3	ev_pop3_1	*cas8*	Frameshift (retention of first 57/435 aa)	No	2,930,725
ev_pop3	ev_pop3_2	CRISPR2 sp1	Deletion	No	2,936,644–2,936,675
ev_pop3	ev_pop3_3	*cas7*	Frameshift (predicted loss of RNA-binding domain)	No	2,927,776
ev_pop3	ev_pop3_4	∆ 60,538 bp	Begins in *cas3* gene and includes CRISPR2 array	Yes	2,934,007–2,994,541
ev_pop3	ev_pop3_5	∆ 34,249 bp	Includes CRISPR region	Yes	2,903,549–2,937,794
ev_pop3	ev_pop3_6	Cas gene deletion	Deletion of *cas7* and *cas5*, partial deletion of *cas6* and *cas8*	No	2,927,288–2,930,093

We observed that the remaining 44% (8/18) of evolved lysogen isolates carried deletions of varying sizes; the smallest being a 3-kb deletion in the *cas* genes, and the largest deletion, 335,331 bp, comprising about 5% of the PA14 chromosome. Seven deletions were centered on the CRISPR2 array (Fig. 2c). These deletions ranged from approximately 34 to 335 kb (mean = 163.3 ± 100.3 kb). Deletions were independent in each isolate, with no shared deletion boundaries even within the same culture (Fig. 2c). Similarly, a previous study showed that PA14, when challenged with free DMS3 virions and subjected to a short-term evolution experiment, evolved genome deletions encompassing the CRISPR region in the presence of self-targeting [see Extended Data Table 1 in reference (44)]. Therefore, in the evolved lysogen populations, we observed extensive coexistence between combinations of CRISPR spacer mutations and large entire deletions of CRISPR, demonstrating the importance of phage infection in determining distinct evolutionary trajectories in isolates in the same environment.

### Evolved lysogens with large deletions are polylysogens

**Fig 3:**
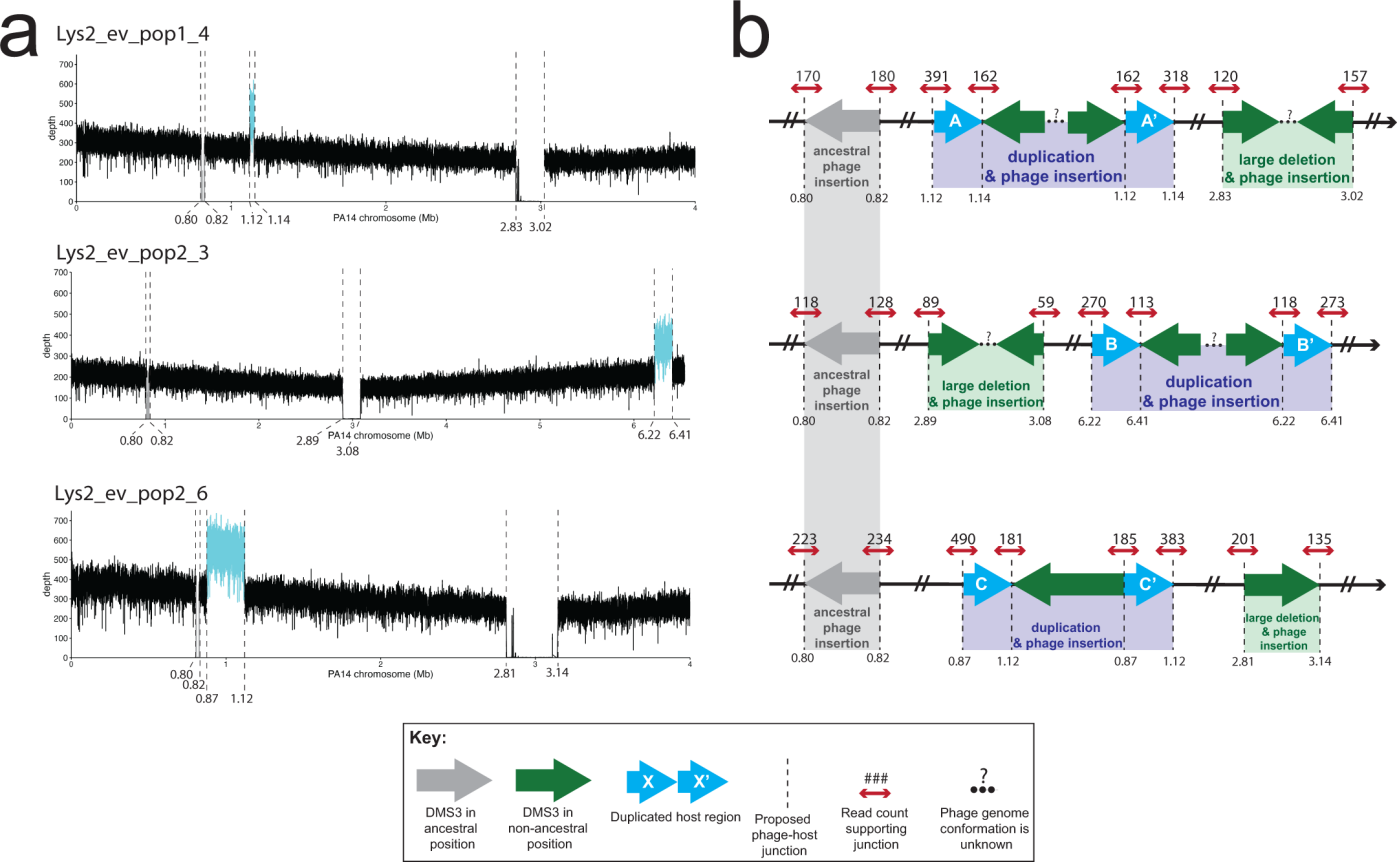
Location and characterization of large duplicated regions in three evolved lysogen isolates. (a) Coverage plots of the host genome. Dashed lines indicate evidence of host-phage boundary. *X*-axis is the position on the PA14 chromosome. Where possible, the *x*-axis has been truncated to view all possible insertion sites. *Y*-axis is the depth of coverage at that nucleotide. Cyan line represents the putative duplicated region in between two viral insertion sites. Gray line is the region between the ancestral insertion sites. Regions of coverage in the deletion region in Lys2_ev_pop1_4 and Lys2_ev_pop2_6 are from domains in a TpsA1 and TpsB1 protein (annotated as a filamentous hemagglutinin protein), and in the 3′ end of the Lys2_ev_pop2_6 deletion, a domain in OprB (annotated as a porin). Both were identified via BLASTX. (b) Cartoon of resulting genome architecture of the evolved lysogens as represented in (a), from top to bottom. Dashed lines indicate a putative host-phage boundary. Gray arrows represent the phage at its ancestral insertion site; green arrows represent phage at new insertion sites. Blue arrows represent duplicate host sequences. The direction of the arrow indicates 5′ to 3′, with 3′ ending at the tip of the arrowhead. Dashed lines represent the junctions between phage and host. Small red arrows indicate host reads; numbers above the arrow indicate read count at that site that supports that junction. Ellipses and question marks between phage genomes represent uncertainty. Read files for each sample and at each location can be found in supplemental Data.

While confirming that the evolved lysogens had retained the phage at its original integration site (Materials and Methods), we found that the boundaries of the deletions of the CRISPR regions were composed of reads that mapped to both the PA14 and DMS3 chromosomes (hereafter referred to as “split” reads), indicating that the large deletions in these seven isolates resulted from a DMS3 transposition event that occurred from within the chromosome (Fig. 2c). Therefore, we consider these deletions to be phage mediated. The regions of the phage chromosome to which the split reads mapped and the orientation of phage reads at the boundaries of the deletions in two samples (1_4 and 2_3) suggest that more than one phage genome may be inserted in the gap (Fig. 3d through e; Table 2). Notably, we did not recover any mutations in the phage chromosome. This work clearly shows that the mechanism of deletion is through phage transposition, which may arise from failed or partial induction events within a lysogen.

**TABLE 2:** Origin of phage reads at the deletion boundaries^*a*^

Sample ID	Group	Upstream deletion boundary Read mate maps to:	Downstream deletion boundary Read mate maps to:
Lys2_ev_pop1_3	ev_pop_1	End of DMS3 genome	Start of DMS3 genome
*Lys2_ev_pop1_4	ev_pop_1	Start of DMS3 genome	Start of DMS3 genome
Lys2_ev_pop2_1	ev_pop_2	End of DMS3 genome	Start of DMS3 genome
*Lys2_ev_pop2_3	ev_pop_2	Start of DMS3 genome	Start of DMS3 genome
Lys2_ev_pop2_6	ev_pop_2	End of DMS3 genome	Start of DMS3 genome
Lys2_ev_pop3_4	ev_pop_3	End of DMS3 genome	Start of DMS3 genome
Lys2_ev_pop3_5	ev_pop_3	End of DMS3 genome	Start of DMS3 genome

^*a*^ Asterisks denote samples where reads from only one end of the phage genome were recovered.

The large deletions, though centered around the CRISPR locus, had different deletion boundaries. To assess the shared gene content in these regions, we used eggNOG-mapper to query the functional protein content that was lost (Materials and Methods). In addition to CRISPR, the deleted regions were enriched with genes from COG category S (genes of unknown function), which included Type 6 secretion system-related genes *tssF* and *tssG*, and multiple pyoverdine system genes, which are often lost during lung colonization (6, 74, 75). Several pyoverdine biosynthesis and transport genes (*fpvA, pvdE, pvdH, pvdI, pvdM, pvdM,* and *pvdO*) were deleted in six of the seven isolates with large deletions. *PvdS,* a regulator of pyoverdine biosynthesis genes (76), and *pvdR,* a component of the pyoverdine efflux transporter (77), were also deleted in five of the seven isolates with large deletions. The co-localization of these virulence and defense gene cassettes with CRISPR may contribute to variation in these regions (78, 79).

### Evolved polylysogens contain large duplications

Three isolates containing phage-mediated large deletions in CRISPR also had large duplications elsewhere in the genome (27, 188, and 244 kb; mean = 153 ± 113 kb). In these cases, these regions were not deleted but were doubled in coverage (Fig. 3a through c). The location of these large duplications exactly corresponded to additional insertion sites, which were recovered by our pipeline (Materials and Methods). Due to the orientation of the PA14/DMS3 split reads at these insertion sites, which faced away from each other rather than toward each other, and due to the fact that the split reads at the boundaries of the duplicated regions only represented about 50% of the total coverage, we interpret these regions to be large duplications with a phage genome in the middle, as opposed to two independent viral insertions (Fig. 3d through f). As the duplicated regions were not centered around a shared core, they were almost completely non-overlapping in their gene content. Only one gene was duplicated in two of the three isolates (2_3 and 2_6), which was *betT,* a choline transporter known to accumulate mutations in clinical isolates from CF patients (80–82). Broadly, genes from category H (coenzyme metabolism) were represented in all three isolates, as well as from P (inorganic ion metabolism) and S (genes of unknown function), as in the large deletions. Several genes annotated as part of the major facilitator superfamily, a class of membrane-associated transporter proteins, were also duplicated in two of the three isolates, as well as genes from the *moa* family, which have recently been implicated in biofilm formation (83). The fact that deletions and duplications caused by transposition are found in the same genome suggests that at least a small number of cells induced phage transposition into multiple regions of the chromosome but did not lyse (84, 85).

We noticed that two of these duplications (in 1_4 and 2_6) independently evolved a shared boundary six nucleotides apart (at positions 1,120,309 and 1,120,303, respectively) in an intergenic region between the 3′ end of a hypothetical protein and the 3′ end of an AraC transcriptional regulator. Accordingly, we investigated whether these new insertion sites shared any sequence similarities. An analysis of all new lysogen insertion sites (e.g., the deletion and duplication boundaries) using the motif-finding software MEME Suite did not return any motifs, either using MEME (searching for a motif in a 15-bp region centered on the insertion site) or MEME-ChIP (searching for centrally enriched motifs 250 bp around the insertion site) (86, 87).

Neither the isolates containing large deletions nor the ones containing large duplications differed in their growth from other evolved strains that did not have large structural variation (Fig. S8A). This suggests that, under these conditions, the fitness costs to deletions, duplications, or carrying additional copies of the phage in the chromosome are smaller than the fitness gains by removing self-targeting. Although these phage-mediated deletions of CRISPR represent the addition of one to two phage genomes to the lysogen chromosome, the spontaneous induction rate of these isolates remains reduced relative to the ancestral PA14 lysogen strain (Lys2) (Fig. 1; Fig. S8B). Additionally, although viral output does not change with phage genome copy number after challenge with mitomycin C, cell survival is significantly increased with increased phage genomes (Fig. S8C), suggesting a possible mechanism of viral interference leading to cell survival, which may also contribute to a decreased spontaneous induction.

### Spontaneous induction correlates with mutation

**Fig 4:**
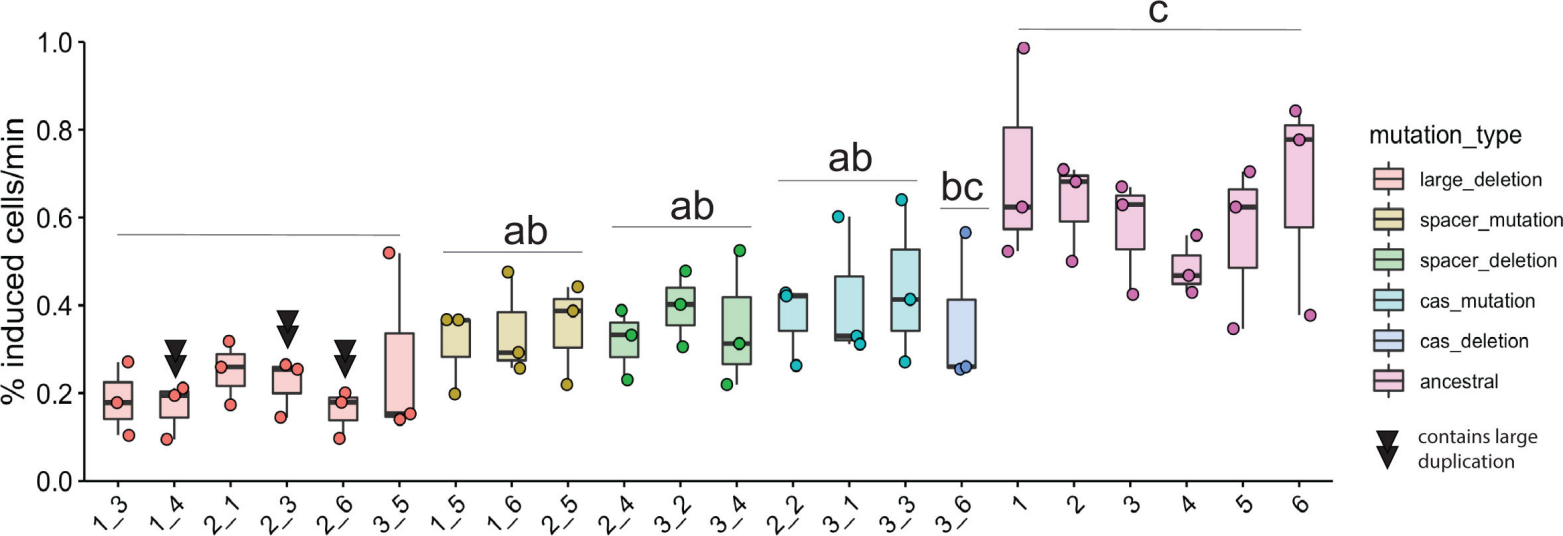
Genomic variation may explain phenotypic variation. Evolved isolates were grouped by type of mutation. Colors of points and boxes represent groups. Points are means of experimental replicates. Box plots are as in Fig. 1. Isolates with double black triangles have large duplications, which are due to another viral insertion. Significance was determined with an ANOVA with Tukey adjustment using the log-transformed values (*F*_5,66_ = 19.3, *P*-value = 8.725e-12). Letters indicate significance; groups with different letters have a *P*-value < 0.05 between them; groups with the same or overlapping letters have a *P*-value of >0.05 between them.

We observed that spontaneous induction was variable among six isolates from each replicate population (Fig. 1). We asked whether this variation might correlate with differences in the type of CRISPR mutation [single nucleotide polymorphisms (SNPs), small deletions, viral transposition, and structural variation]. To address this, we grouped lysogens into one of five categories based on the type of mutation that occurred in the genome (“cas deletion”; “cas mutation”; “spacer deletion”; “spacer mutation”; and “large deletion polylysogen”) and asked whether including mutation type explained the variation within these groups. We observed that all groups (with the exception of the cas deletion group, which had only one isolate in its group) had significantly lower spontaneous induction than the ancestral strains (Fig. 4), and the large deletion polylysogen group was significantly lower than lysogens that had lowered spontaneous induction via SNPs or indels (Fig. 4, ANOVA, *F*_5,66_ = 19.3, *P*-value = 8.725e-12). Another set of experiments that included a lysogenized ∆CRISPR strain showed that evolved lysogens, which resolved genetic conflict via genome rearrangements, SNPs, and indels, reduced CRISPR function to the level of a ∆CRISPR mutant (Fig. S9). A model incorporating mutation type was a significantly better fit than the model by experimental replicate (Fig. 4, ANOVA, *F*_2,66_ = 13.777, *P* < 1e-6). In view of these results, we find that heterogeneity in mutation type correlates to the heterogeneity in phenotype of spontaneous induction in our evolved lysogens. Although large deletions had the lowest spontaneous induction and therefore the highest fitness, they did not dominate any of the three populations.

**Fig 5:**
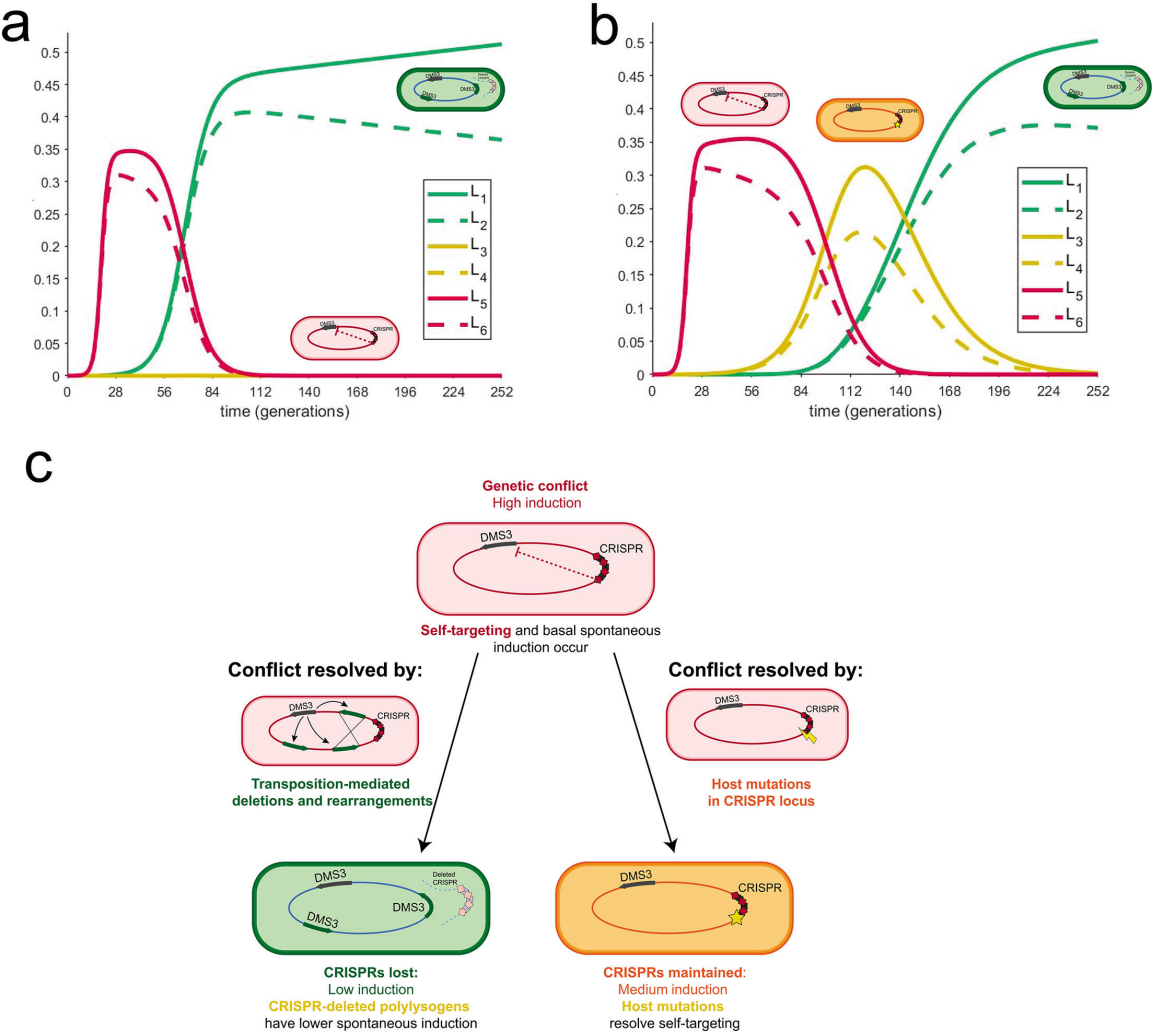
Coexistence of different rates of spontaneous induction depends on the order of their introduction. (a) Mathematical model describes the behavior of several lysogens with different spontaneous induction values. These graphs describe the growth of six lysogens over time in one continuously growing culture. The *y*-axis represents the proportion of carrying capacity of the medium; the *x*-axis is time in PA14 generations. Each lysogen was assigned a spontaneous induction value from the experimentally determined range. Each pair (L1/L2; L3/L4; and L5/L6) in a certain range is taken from values in the same group, from lowest to highest spontaneous induction values (L1 is the lowest; L6 is the highest). In panel a, the highest spontaneous inducers are introduced first. At approximately 9 hours, low inducers are introduced; after about 1 day, medium inducers are introduced and do not establish. In panel b, the highest spontaneous inducers are introduced first. At approximately 9 hours, medium inducers are introduced; after about 1 day, low inducers are introduced and establish, resulting in a period of coexistence between medium and low inducers that is observed experimentally. (c) A model of self-targeting resolved by different modes of mutation. Spontaneous induction caused by CRISPR self-targeting is resolved in two modes. Cells with high spontaneous induction are indicated in red. One mode relies on low levels of DMS3 escape from lysogenic repression, which results in rare transposition events around the genome. Recombination between multiple DMS3 chromosomes leads to large deletions, which may include the CRISPR area and results in cells with lower spontaneous induction (green). In the second mode, self-targeting may be resolved by host mutations in the CRISPR-Cas array (orange) or by viral mutations in the targeted region (not recovered in this study). Integrated DMS3 genomes are represented with arrows to indicate directionality.

Given these small but significant variations in spontaneous induction that are maintained within groups yet replaced the ancestral lysogen genotype, we wanted to understand how long the diversity we observed within our experimental replicates could persist in exponentially growing cultures. To do this, we developed a mathematical model to compare six lysogens with six different rates of spontaneous induction (two values from the ancestral group, two values from the host-mutation group, and two values from the large deletion polylysogen group). In a deterministic model of lysogen growth in the exponential phase with varied spontaneous induction rates, we found populations with low densities of high inducers (representing the ancestor strain) and allowed them to grow for 10 hours. After 10 hours, we introduced either strains with low rates of spontaneous induction (representing the large deletion polylysogens) or medium rates of spontaneous induction (representing host mutations), one at 10 hours and the other at 24 hours, and tested whether they could invade. We found that when low inducers are introduced to a high-inducing population, medium inducers cannot subsequently invade (Fig. 5a). When we introduced medium inducers to a high-inducer population after 10 hours of growth and then low inducers after 24 hours, medium and low inducers outcompeted the high inducers and then coexisted in the absence of the high inducers for about 2 days (Fig. 5b). This coexistence between low and medium inducers recapitulates the observed recovery of low and medium inducers, but not high inducers, in our experimental data (Fig. 4; for a model, see Fig. 5c). Given the higher fitness, but not a complete takeover, of large deletion polylysogens, we infer that deletions introduced by transposition occur at a lower rate than spontaneous mutation. From these observations, we find that the weak selection imposed by these small differences in spontaneous induction, which are caused by different mutational mechanisms, combined with the order in which they were introduced, may allow the evolution of diversity in CRISPR self-targeting resolution in *P. aeruginosa* lysogens and preserve coexistence of CRISPR+ and CRISPR− strains in the absence of additional selective variables or environmental change.

## DISCUSSION

Although temperate phages are frequently recovered from long-term, evolving *Pseudomonas* infections of the lungs of CF patients, phages are often considered at single time points or outside of relationship to their bacterial hosts (18, 88–90). Evolution experiments involving transposable phages often include susceptible cells or are begun with free phages instead of established lysogens (42–44, 91). Because these transposable phages may insert into the chromosome in many different locations, their frequency in the population as lysogens is defined by selection for beneficial bacterial mutations. Here, we show that this occurs in established PA14-DMS3 lysogens where ongoing transposition results in polylysogens that are selected for because they resolve genetic conflict by causing structural variants. This additional mutational mode, which occurs in addition to the host mutation rate, introduces different types of variation that coexist within a once-clonal population.

Our work supports previous studies showing that DMS3 lysogens evolve decreased rates of spontaneous induction over time (44, 51). Here, we distinguish between exponential and stationary phase and find that exponential phase induction accounts for the majority of free phages in the medium. Our spontaneous induction estimates in the exponential phase are approximately 0.37% and 0.72% of the evolved and ancestral populations, respectively, which are both significantly higher than lambda-like phages (57, 92, 93) but are in line with other studies that report high spontaneous induction (94). Mu-like phages have been reported previously to naturally produce high titers during lysogen growth (26, 45, 51), suggesting that this evolutionary pressure is not restricted to DMS3 and that Mu-like phages as a family have high spontaneous induction rates. Because Mu-like phages are both highly prevalent and highly targeted in *P. aeruginosa* (49), it is likely that CRISPR self-targeting will inform evolutionary outcomes in lysogens of these related phages. Here, the resolution of self-targeting resulted in coexisting variation in lysogen spontaneous induction rates in exponential growth, with polylysogens having the lowest rates, but they are rare as a mutational class. Though rare, these deletions, perhaps formed through incomplete or partial induction (84, 85), would likely fix in a population that was evolved for longer than 12 days.

We find that the genetic conflict between Mu-like phage and host results in a tradeoff between CRISPR immunity and spontaneous induction, which could help explain the maintenance of CRISPR systems in *P. aeruginosa* (95–97). Previously, mutations in *cas7* (51) and deletions of the entire CRISPR region (44) have been found to reduce phage induction in the late log phase. We find that these differences in self-targeting resolution reduce phage induction to different degrees and have profoundly different effects on the host genome. Half (10/18) of the evolved lysogen isolates decreased their spontaneous induction while maintaining either CRISPR function (in mutations or deletions of the self-targeting spacer) or the potential of CRISPR function (frameshift mutations in *cas* genes), while the rest deleted CRISPR and lost any potential immunity, but more substantially decreased their induction. Although it is possible that CRISPR targeting directly induces transposition via some interaction of CRISPR machinery with DMS3, our model for the generation of variation does not require this. Instead, our model requires transposition from within the lysogen, which then causes variation through deletion and duplication but does not result in lysis. Polylysogens from the coliphage Mu have only rarely been isolated after low levels of induction: once due to a recombination event (85), and once after surviving partial heat induction (84). Our results suggest that stochastic transposition occurs at low rates relative to mutation but is recovered here because of selection for the large deletions, which lead to lower spontaneous induction.

One explanation for the coexistence of these two genotypes (CRISPR maintenance and higher induction, or CRISPR loss via polylysogeny and lower induction) is the order in which these mutations were introduced. Modeling simulations show that spontaneous induction rates from strains that resolved self-targeting via SNPs and indels (“medium” inducers) do not invade established populations of strains with spontaneous induction rates from polylysogens (“low” inducers). This indicates that host-mediated SNPs and indels likely arose before polylysogeny and large deletions, as both are maintained together despite spontaneous induction differences. This suggestion of an order (host-mediated before phage-mediated) then suggests that the basal rate of phage transposition is lower than host mutation. A lower rate of formation of higher-fitness polylysogen “low” inducers, which compete with lower-fitness host-mutation “medium” inducers with a faster rate of formation, may work to maintain a pool of diversity that selection may subsequently act on in different ways, given the presence of other phages or other ecological factors (98).

The results of genetic conflict in evolved lysogens are not limited to deletions of CRISPR and may impact the rate of evolution of bacteria with latent infections. Gene loss and genome reduction have also been shown to occur in *P. aeruginosa* lineages during adaptation to the human lung, although the contribution of phages to this loss is unclear (13, 89). In this study, pyoverdine and Type 6 secretion system genes were lost in the majority of the polylysogens, and one evolved lysogen isolate (1_1) had a nonsynonymous mutation in the Type 6 secretion system baseplate gene *tssK* (Table 1). These genes, lost under laboratory conditions, are also often lost in chronic CF isolates (75, 81, 99).

The mechanism of large deletion formation is unclear in two isolates. In most (5/7) evolved polylysogens, a phage genome simply replaced the deleted sequence as in reference (44). In these isolates, the phage genome appeared in a head-tail configuration, which could occur as a result of the replicative transposition reaction itself or as a result of recombination between two preexisting phages. Recombination between a duplicated sequence is an attractive hypothesis to explain the deletions because it may lead to deletion or duplication of the intervening sequence (100). However, two isolates, which both contain a duplication (1_4 and 2_3), exhibit non-canonical deletion and duplication structures, where we recover host-phage junctions, which suggest two phage genomes facing either head-head (reads recovered at both junctions which map to the 5′ end of the genome) or tail-tail (reads recovered at both junctions which map to the 3′ end of the phage genome). Additionally, recombination may result in two phage-host junctions on the 3′ end of the duplication, which lead in different ends of the phage chromosome, whereas we only recover reads that lead in one end of the phage chromosome (101).

An alternative model of large deletion formation is one in which primed spacer acquisition of chromosomal spacers leads to CRISPR loss to avoid self-targeting-induced cytotoxicity. Primed spacer acquisition can occur from degraded spacer-protospacer pairs, which have extensive mismatches (102). Acquisition of self-targeting spacers by Cas1 could lead to Cas3-mediated DNA damage and subsequent deletions (103). However, we show that every deletion boundary is composed of reads that map to the phage, indicating that phage transposition from within an established lysogen, and not deletions that can be generated from new infections, is sufficient to generate these genome rearrangements. It has been previously demonstrated that self-targeting can select for the spontaneous deletion of the targeted element (104, 105), which we did not observe, likely because of protection from phage curing by free viruses constantly present in the lysogen media through superinfection exclusion (106). Whether and how phage presence continues to alter the evolution of its host from the uninfected state and how phage infection influences the rate and mechanism of this evolution are questions that require future study to explain the ubiquity of phage infection in many clinical environments (15, 107, 108).

*P. aeruginosa* evolution in the context of the CF lung can occur via a slow accumulation of SNPs and indels (81) or a more rapid accumulation of SNPs due to the evolution of hypermutator genotypes (81, 109). Lysogenization by transposable phages may offer a different mechanism of within-lung diversification, which operates in addition to the baseline mutation rate. Due to the nature of short-read sequencing, it is likely that polylysogeny of the same virus, and resulting genome rearrangements, have been under-represented in current data sets of *P. aeruginosa* clinical isolates. Future studies should continue to identify signatures of multiple phage infections in clinical isolates and look for deletions and duplications that may be associated with phages. Polylysogeny of transposable phages may contribute to understanding variation in CRISPR carriage among bacteria, although if polylysogeny is accompanied by CRISPR deletions, it may be difficult to ascertain in which background they arose. The potential link between transposition and low levels of DNA damage also opens the possibility of other causes of low levels of DNA damage—for example, subinhibitory concentrations of DNA-damaging antibiotics—to be driving evolutionary adaptation and diversity in bacteria lysogenized by transposable phages. The extent to which phage infection informs differences in evolutionary and functional outcomes in a clinical context is an important subject for future work.

## Data Availability

All raw reads are available on the NCBI database under BioProject number PRJNA1021667. Data and code for figures are available at https://github.com/lsuttenfield/mSystems-2024.

## AFTER EPUB

*[This article was published on 21 August 2024 with an error in Fig. 5. The figure was corrected in the current version, posted on 27 August 2024.]*

## SUPPLEMENTAL MATERIAL

Supplemental material msystems.00801-24-s0001.docx Supplemental figures and tables.

ASM does not own the copyrights to Supplemental Material that may be linked to, or accessed through, an article. The authors have granted ASM a non-exclusive, world-wide license to publish the Supplemental Material files. Please contact the corresponding author directly for reuse.
