# Supplementary material for: Phage-mediated resolution of genetic conflict alters the evolutionary trajectory of Pseudomonas aeruginosa lysogens
Source: mSystems. 2024 Aug 21;9(9):e00801-24. doi: 10.1128/msystems.00801-24 (PMC11406979; doi:10.1128/msystems.00801-24)
Supplement: Supplemental material — Supplemental figures and tables. [file msystems.00801-24-s0001.docx]

**Supplemental Figures**

**Figure S1. Uninfected populations do not share mutations with lysogen populations.** The Y-axis indicates sample ID; X-axis indicates position on the PA14 reference chromosome. Circles represent point mutations; triangles spanned by a segment represent deletions of the spanned region; squares spanned by a segment represent duplications of the spanned region. Inset white shapes indicate a mutation that was caused by a virus. Uninfected population PA14_ev_pop2 contains evidence of phage selection pressure as 100% of isolates have a mutation in the Type 4 pilus. Lysogen data is the same as in Figure 2.

**
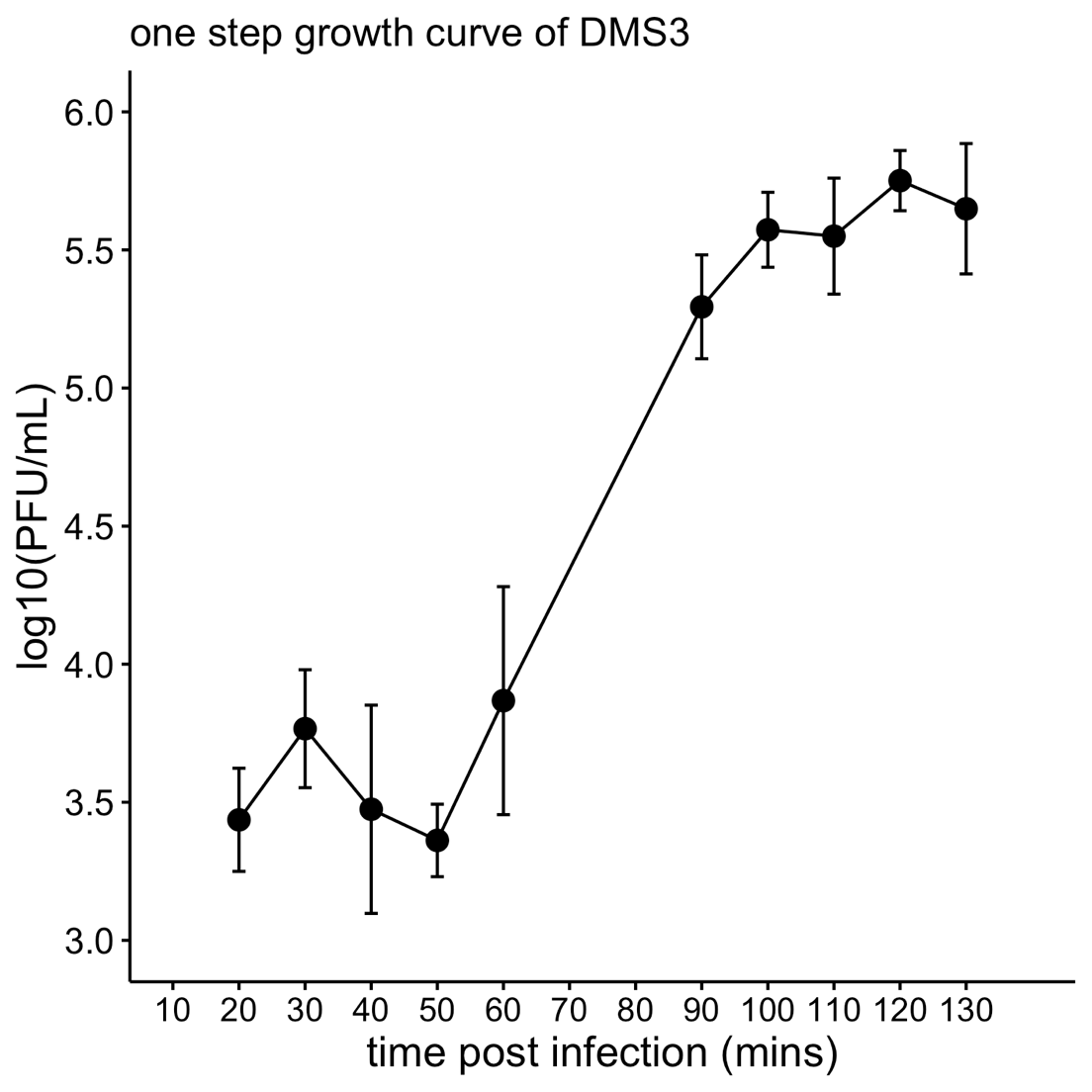
**

**Figure S2. One step growth curve of DMS3**. Points represent the mean of experimental replicates (n=6 for all other time points except 20 mins (n=3) and 100 mins (n = 2). Error bars represent standard deviation. The x-axis is time post-infection in minutes; the y-axis is the log_10_ of PFU/mL. Plateau 1 is the average of all values from 20-50 minutes; Plateau 2 is the average of all values from 100-130 minutes.

**Figure S3. Spontaneous induction calculated with Zong et al formula.** A) Spontaneous induction in exponential phase. X-axis is time in hours; y-axis is spontaneous induction per hour, or 1.6 cell generations of PA14. B) Spontaneous induction in stationary phase. X-axis is sample ID; y-axis is spontaneous induction per hour. Significance was calculated as the spontaneous induction value as a function of group with Dunn's Test of Multiple Comparisons and Bonferroni correction. Letters indicate significance; groups with different letters have a p-value <0.05 between them; groups with the same or overlapping letters have a p-value of >0.05 between them.

**Figure S4. Experimental evolution results in lowered lysogen spontaneous induction in stationary phase.** Spontaneous induction was measured in stationary phase. Six individual isolates from each of three lysogen replicates (ev_pop_1, pink), (ev_pop_2, blue), (ev_pop_3, light blue) and the ancestral strain Lys2 (anc_pop_2, grey) were measured. Points are the means of one experimental replicate from three technical replicates. Boxplots are as in Figure 1. Significance was tested with an ANOVA (*F*_3,109_ = 86.98, *P* < 2.2e-16). Letters indicate significance; groups with different letters have a p-value <0.05; groups with the same or overlapping letters have a p-value of > 0.05.

**Figure S5. DMS3 is partially matched by CRISPR spacers in PA14 and is resolved by host mutations.**  Map of spacer-protospacer match compared to escape mutations in the host spacer that evolved in two single isolates in parallel cultures. Five mismatched bases on the virus are highlighted in bold red font and lack lines indicating base pairing; escape mutations are highlighted in blue and lack lines indicating base pairing. Hyphens indicate a deletion of those bases.

**Figure S6. No new spacer acquisition, only spacer evolution in the CRISPR2 array after lysogen evolution.** We ran CCTK on the evolved lysogen genomes to look for spacer acquisition. CCTK only recovered known spacers. A CCTK graphical output representing all different arrays from these genomes. Spacers shared between arrays are denoted by boxes with the same colored outline and fill. Spacers that are unique only to that array and which are not shared with any other array are represented as a circle, triangle, and star. In our case, these unique spacers are due not to spacer acquisition but to point mutation which make them non-identical and “unique” with respect to the others. Three cases of unique spacers are due the representation of the ancestral spacer (maintained in all strains which resolve self-targeting via the cas genes) and two unique spacer genotypes.


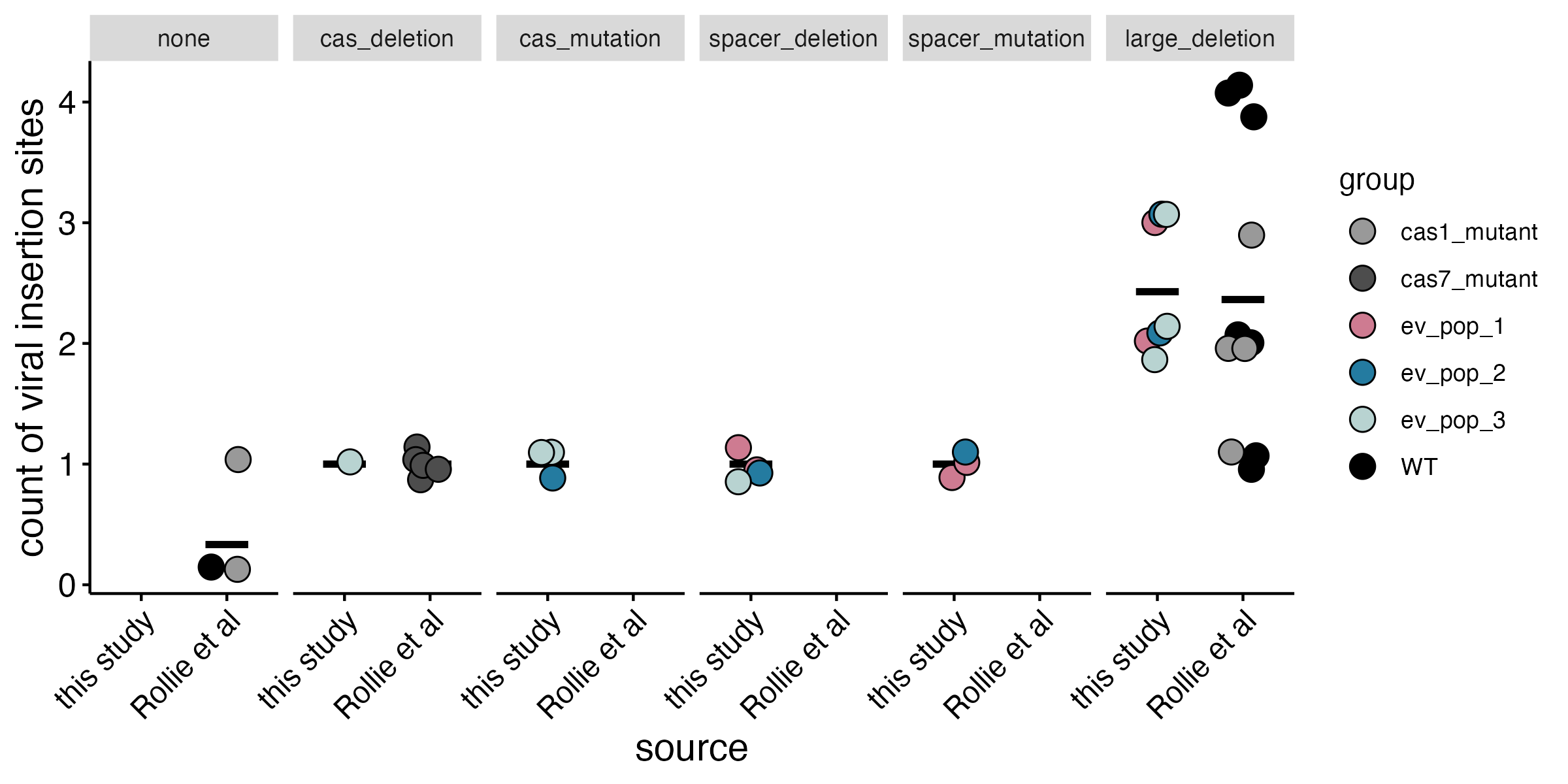


**Fig S7. Polylysogeny is not selected for in the absence of self-targeting.** Y-axis describes the number of viral insertion sites recovered from each isolate; X-axis describes the source of the isolates (either this study or Rollie et al) and is broken up by the type of mutation which the isolate is classified by. Black bars indicate the mean. Points represent one isolate. Point color indicates either what replicate it came from (as in this study) or in what strain background the evolution experiment was started (in Rollie et al). NA: no isolates were recovered in that mutation type category.


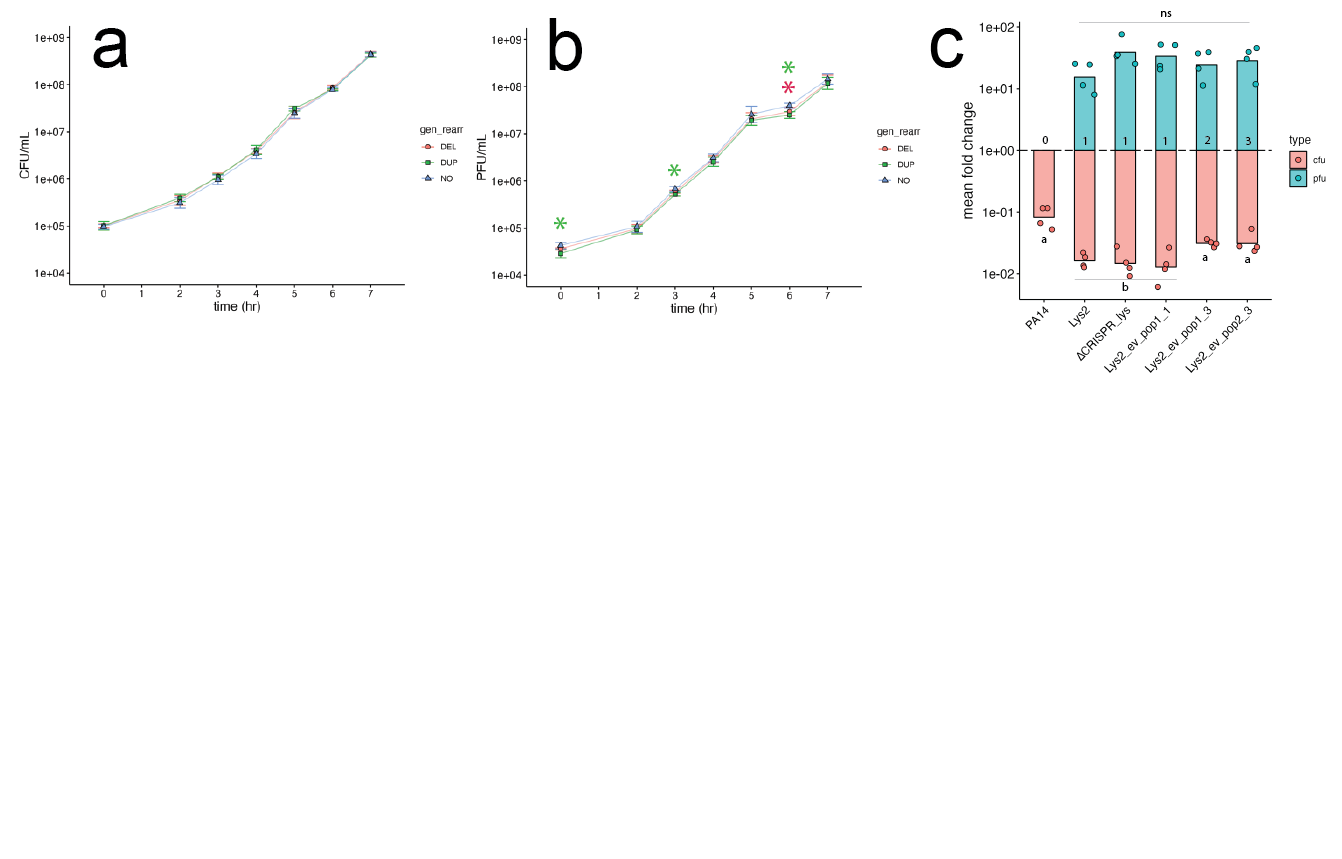


**Figure S8. Genome rearrangements are tolerated without growth defects in rich medium in evolved lysogens.** A) CFUs and B) PFUs of evolved lysogens in exponential growth. Time is indicated on the x-axis in hours, and CFUs or PFUs are indicated on the y-axis. Asterisks indicate significant differences by Dunn’s test between the genomic rearrangement (“DEL”: deletion; “DUP”: duplication) indicated by the color, and the “NO” genomic rearrangement group (the average of all other lysogens). C) Induction of lysogens with increasing numbers of viral genomes. Numbers in the PFU column represent how many viral genomes are present in the lysogen. Dunn’s Test of Multiple Comparisons with a Bonferroni correction was used to test the interaction between the mean fold change and the number of phages present. Points represent the mean of one experimental replicate (three technical replicates each). Bars represent the mean of all experimental replicates. Letters indicate statistical significance between groups; groups with the same letter are not statistically significant. “NS” = not significant.

**Figure S9. Spontaneous induction differences are maintained between groups and ∆CRISPR lysogen.**  Spontaneous induction was measured as above, except the growth curve was begun with a 1:100 diluton instead of 1:1000 (OD600 = 0.002). All differences between mutation groups was recovered, except the cas deletion group became significantly different from the ancestral, and the large deletion polylysogen group became significantly different from the spacer mutation group. Significance was tested with an ANOVA (*F_6_*_,110_ = 28.35, *P* < 2.2e-16) with a Tukey adjustment. Letters indicate significance; groups with different letters have a p-value <0.05; groups with the same or overlapping letters have a p-value of > 0.05. Points are the means of one experimental replicate from three technical replicates. A small jitter was added to the horizontal to increase visibility. Bars in the boxplots represent the median; crosses represent the means. Upper and lower bounds of the box are the upper and lower interquartile ranges.

| **Strain** | **Description** | **Source** |
| --- | --- | --- |
| PA14 | WT P. aeruginosa strain | George O’Toole |
| Lys2 | PA14(DMS3) lysogen | Zegans et al, 2009 |
| PA14∆CRISPR | CRISPR deletion | Cady et al, 2011 |
| PA14∆CRISPR(DMS3) | CRISPR deletion lysogen | Cady et al, 2011 |

**Table S1. List of strains used in this study.**

| Lys2 Deletion Boundaries: 806165-826108 on PA14 Reference Genome | | |
| --- | --- | --- |
| **Gene no. (5’-3’)** | **Deleted gene** | **Description** |
| 1 | PhzG | phenazine biosynthesis FMN-dependent oxidase |
| 2 | PhzF | phenazine biosynthesis protein; trans-2,3-dihydro-3-hydroxyanthranilate isomerase |
| 3 | PhzE | phenazine biosynthesis protein |
| 4 | PhzD | phenazine biosynthesis protein |
| 5 | PhzC | phenazine biosynthesis protein; phospho-2-dehydro-3-deoxyheptonate aldolase |
| 6 | PhzB | phenazine biosynthesis protein |
| 7 | PhzA | phenazine biosynthesis protein; |
| 8 | PhzM | phenazine-1-carboxylate N-methyltransferase |
| 9 | OpmD | multidrug efflux transporter outer membrane subunit; TolC family protein |
| 10 | MexI | MexW/MexI family multidrug efflux RND transporter permease subunit |
| 11 | MexH | MexH family multidrug efflux RND transporter periplasmic adaptor subunit |
| 12 | MexG | DoxX family protein; multidrug efflux RND transporter inhibitory subunit |
| 13 | PpgL | Gluconolactonase; 3-carboxymuconate cyclase |
| 14 | NmoR | LysR family transcriptional regulator |
| 15 | NmoA | nitronate monooxygenase |
| 16 | - | D-alanine--D-alanine ligase |
| 17 | - | MBL fold metallo-hydrolase |

**Table S2. Genes contained in Lys2 deletion.** All functional predictions were confirmed by BLASTX using the protein RefSeq database.

| Group | Sample ID | Mutated region | | Description | Location on PA14-REF chromosome |
| --- | --- | --- | --- | --- | --- |
| ev_pop1 | ev_pop1_1 | | *fliF* | Nonsense mutation in flagellar M-ring protein _G→A_ | 4459793 |
| ev_pop1 | ev_pop1_2 | | *fliP* | Nonsynonymous mutation in start codon in flagellar biosynthesis protein _C→T_ | 4072428 |
| ev_pop1 | ev_pop1_3 | | *fliF* | Nonsense mutation in flagellar M-ring protein _G→A_ | 4459793 |
| ev_pop1 | ev_pop1_4 | | *fliF* | Nonsense mutation in flagellar M-ring protein _G→A_ | 4459793 |
| ev_pop1 | ev_pop1_5 | | *lasR* | Nonsynonymous mutation in transcriptional activator _C→T_ | 4088064 |
| ev_pop1 | ev_pop1_6 | | *fliF* | Nonsense mutation in flagellar M-ring protein _G→A_ | 4459793 |
| ev_pop1 | ev_pop2_1 | | *pilY1* | Frameshift mutation in T4P biogenesis factor protein ­_GG→G_ | 5376474 |
| ev_pop1 | ev_pop2_2 | | *pilY1* | Frameshift mutation in T4P biogenesis factor protein ­_GG→G_ | 5376474 |
| ev_pop1 | ev_pop2_3 | | *lasR* | Nonsynonymous mutation in transcriptional activator _C→T_ | 4088064 |
| ev_pop2 | ev_pop2_3 | | *pilY1* | Nonsynonymous mutation in T4P biogenesis factor protein _T→A_ | 5378668 |
| ev_pop2 | ev_pop2_4 | | *lasR* | Nonsynonymous mutation in transcriptional activator _G→A_ | 4088043 |
| ev_pop2 | ev_pop2_4 | | *pilY1* | Frameshift mutation in T4P biogenesis factor protein ­_GG→G_ | 5376588 |
| ev_pop2 | ev_pop2_5 | | *lasR* | Nonsynonymous mutation in transcriptional activator _C→T_ | 4088064 |
| ev_pop2 | ev_pop2_5 | | *pilY1* | 1kb deletion in T4P biogenesis factor protein | 5377966 |
| ev_pop2 | ev_pop2_6 | | *lasR* | Nonsynonymous mutation in transcriptional activator _G→A_ | 4088043 |
| ev_pop2 | ev_pop2_6 | | *pilY1* | Frameshift mutation in type 4 pilus biogenesis factor protein ­_GG→G_ | 5376588 |
| ev_pop2 | ev_pop3_1 | | *fliF* | Nonsense mutation in flagellar M-ring protein _G→A_ | 4459793 |
| ev_pop2 | ev_pop3_2 | | *fliP* | Nonsynonymous mutation in start codon in flagellar biosynthesis protein _C→T_ | 4072428 |
| ev_pop3 | ev_pop3_2 | | MFS transporter | Synonymous mutation _G→A_ | 4170934 |
| ev_pop3 | ev_pop3_3 | | *fliF* | Nonsense mutation in flagellar M-ring protein | 4459793 |
| ev_pop3 | ev_pop3_4 | | *No mutations* | *NA* | *NA* |
| ev_pop3 | ev_pop3_5 | | C4 RNA | Point mutation in predicted C4 RNA | 4288779 |
| ev_pop3 | ev_pop3_6 | | C4 RNA | Point mutation in predicted C4 RNA | 4288779 |

**Table S3. Description of mutations recovered in evolved uninfected PA14.**

**Supplemental Information**

The following parameter values were used in the model equations:

$$\frac{dL_{i}}{dt}=rL_{i}\left( 1-\sum_{j=1}^{6} \left( L_{j}+I_{j} \right) \right)-q_{i}L_{i}$$

$$\frac{dI_{i}}{dt}=-\delta I_{i}+q_{i}L_{i}$$

$\frac{dV_{i}}{dt}=\beta\delta I_{i}-\mu V_{i}$.

$$q_{1}=0.1, q_{2}=0.102, q_{3}=0.17, q_{4}=0.175, q_{5}=0.288, q_{6}=0.293$$

and

$$r=1, \beta=45, \delta=4,\mu=1.$$

Each generation lasts 38 minutes. The initial conditions used were $\frac{1}{6}\times{10}^{-4}$ for each $L_{i}$, which was added at the prescribed times. $I_{i}$ and $V_{i}$ were initially zero for each $i=1,\ldots, 6.$
